# Supplementary material for: A deep learning framework for the early detection of multi-retinal diseases
Source: PLoS One. 2024 Jul 25;19(7):e0307317. doi: 10.1371/journal.pone.0307317 (PMC11271906; doi:10.1371/journal.pone.0307317)
Supplement: Attachment — Submitted filename: Response to Reviewers.docx [file pone.0307317.s001.docx]

**Rebuttal Letter**

**Date:**	June 27, 2024

**To:**	Muhammad Mateen (Academic Editor)

**From:**	Dr. Zeeshan Ashraf (Corresponding Author)

**Subject:	Revised submission of the manuscript with comments incorporated**

**Manuscript Code Number:** PONE-D-24-19529
**Manuscript Title:** A Deep Learning Framework for the Early Detection of Multi-Retinal Diseases

**Dear Muhammad Mateen,**

Thank you for inviting us to submit a revised draft of our manuscript entitled “A Deep Learning Framework for the Early Detection of Multi-Retinal Diseases” to your journal. We also appreciate the time and efforts you and each reviewer have dedicated to providing insightful feedback on ways to strengthen our research paper. Thus, it is with great pleasure that we resubmit our article for further consideration. We have incorporated changes that reflect the detailed suggestions you have graciously provided. We hope our modifications and the responses satisfactorily tackle all the issues and suggestions the esteemed reviewers have provided.

To facilitate the reviewer, the following is a point-by-point response to the questions and comments in your letter dated **Wednesday, 12 June 2024**.

**Journal Requirements:**

1. **Please ensure that your manuscript meets PLOS ONE's style requirements, including those for file naming.**

The manuscript meets PLOS ONE’s style requirements.

2. **Please note that PLOS ONE has specific guidelines on code sharing for submissions in which author-generated code underpins the findings in the manuscript.**

Yes. Code is available and shared without restrictions.

3. **Thank you for stating the following financial disclosure. Please include this amended Role of Funder statement in your cover letter; we will change the online submission form on your behalf.**

The role of the authors has been added to the manuscript and mentioned in the cover letter.

4. **Thank you for stating the following in the Acknowledgments Section of your manuscript: We note that you have provided funding information that is not currently declared in your Funding Statement. However, funding information should not appear in the Acknowledgments section or other areas of your manuscript. We will only publish funding information present in the Funding Statement section of the online submission form.**

Acknowledgments have been revised in the manuscript and mentioned in the cover letter.

5. **Please provide a complete Data Availability Statement in the submission form, ensuring you include all necessary access information or a reason for why you are unable to make your data freely accessible. If your research concerns only data provided within your submission, please write "All data are in the manuscript and/or supporting information files" as your Data Availability Statement.**

The Data Availability Statement has been included in the manuscript.

6. **We note that Figure 2 in your submission contains copyrighted images. All PLOS content is published under the Creative Commons Attribution License (CC BY 4.0), which means that the manuscript, images, and Supporting Information files will be freely available online, and any third party is permitted to access, download, copy, distribute, and use these materials in any way, even commercially, with proper attribution. We require you to either (1) present written permission from the copyright holder to publish these figures specifically under the CC BY 4.0 license, or (2) remove the figures from your submission:**

Figure 2 has been removed from the manuscript.

**Reviewers' comments:**

**Reviewer #1:**

**1. It is not clear what gap your study has addressed by comparison with other retinal classification methods: apart from augmentation, what do the behavioral features represent (lines 464-468)?**

Thank you for mentioning this point. The research gap that this article has covered is mentioned in lines 501-515 on page number 20/24. The comparison with other methodologies in Literature is updated in Table 12 in page number 19/24.

**2. A discussion of the reasons for choosing these CNN architectures (12 layers, 15 layers, 20 layers) is needed.**

Thank you for highlighting this point. The reasons for choosing these CNN architectures (12 layers, 15 layers, 20 layers) is mention in lines 295-303 in page number 9/24.

**3. Regarding the processing of the data set, the authors stated that "Each image is assigned to a single disease class, rather than having multiple labels." So in the case of retinal images, one image could have multiple diseases. Why didn't you consider a multi-label (multi-output) classification problem then?**

Thank you for addressing this point. The reason for choosing the single label image is mentioned in lines 215-223 in page number 6/24 and reason for multi-label (multi-output) classification problem is mentioned in line 532-535 in page number 20/24.

**4. It is not clear how transfer learning was used? What is the pre-trained architecture used?**

Thank you for pointing this issue. It was a draft mistake as transfer learning is not used in this article. That’s way removed this section.

**5. How did you ensure independence between the training and testing datasets (eg, an original image can be used for training and its augmented variants can be used for testing)? Can the results be influenced in this case?**

Thank you for suggesting this idea. While using the augmentation technique original as well as augmented data is used for training and testing as mention in lines 203-210 in page number 6/24. The experiment is also performed using original data for training and augmented data for testing. When Experiment was performed 471 images for Diabetic Retinopathy, 334 for Media Haze, 172 for Optic Disc Cupping and 931 for Healthy class were used for training. For testing, 20% of the original images from each class were used, along with the augmented data. The Model shows overfitting as shown in Graph given below.

|   (a) |   (b) |
| --- | --- |
|  |  |

**Reviewer #2:**

**1.** **Minor grammar mistakes. Between lines 366-373, two sentences are repeated. The manuscript is mostly free of grammatical errors. However, minor issues such as subject-verb agreement and punctuation should be reviewed.**

Thank you for reading the manuscript thoroughly. The repetition error has been resolved. Proofreading has resolved the subject-verb issue.

**2. Regularization techniques missing. This is the reason why your network is sometimes overfitting and why your accuracy curves are not smooth (and have a zig-zag pattern instead). Batch normalization can be added to the network.**

Thank you for mentioning this issue. Now, experiments are performed by adding regularization technique L2 Regularization, increasing the Dropout Rate from 40% to 50%, and added Data Augmentation to reduce overfitting as mentioned in Tables 2 and 4 (in manuscript page number 8/24 and 10/24). Graphs with augmented data are updated as shown in Figures 8, 11, and 14 (in manuscript in page number 14/24, 16/24, 17/24). In this scenario, Batch Normalization does not reduce the overfitting issue as shown in the graph for CNN-3 (20 Layer CNN) (in manuscript). Some other experiments are also performed with Batch Normalization to check its behavior for this scenario the model shows overfitting as given below.

|   (a) |   (b) |
| --- | --- |
|  |  |

**3. Limited comparison with state-of-the-art. Although there is a comparison with some existing works, the study could benefit from a more extensive comparison with state-of-the-art models in retinal disease detection. This would provide a clearer context for the contribution and significance of the proposed framework.**

Thank you for assisting. The comparison Table 12 is updated and added data in lines 478-488 page number 19/24.

***Thank you very much to the editor and the reviewers for your precious suggestions！***

Sincerely,

**Dr. Zeeshan Ashraf**
